# Present and Future of Anti-Glioblastoma Therapies: A Deep Look into Molecular Dependencies/Features

**DOI:** 10.3390/molecules25204641

**Published:** 2020-10-12

**Authors:** Hyeon Ji Kim, Do-Yeon Kim

**Affiliations:** 1 Department of Pharmacology, School of Dentistry, Kyungpook National University, Daegu 41940, Korea; guswl1634@naver.com; 2 Department of Pharmacology, School of Dentistry, Brain Science and Engineering Institute, Kyungpook National University, Daegu 41940, Korea

**Keywords:** glioblastoma, molecular pathogenesis, immunotherapy, drug resistance, personalized therapies

## Abstract

Glioblastoma (GBM) is aggressive malignant tumor residing within the central nervous system. Although the standard treatment options, consisting of surgical resection followed by combined radiochemotherapy, have long been established for patients with GBM, the prognosis is still poor. Despite recent advances in diagnosis, surgical techniques, and therapeutic approaches, the increased patient survival after such interventions is still sub-optimal. The unique characteristics of GBM, including highly infiltrative nature, hard-to-access location (mainly due to the existence of the blood brain barrier), frequent and rapid recurrence, and multiple drug resistance mechanisms, pose challenges to the development of an effective treatment. To overcome current limitations on GBM therapy and devise ideal therapeutic strategies, efforts should focus on an improved molecular understanding of GBM pathogenesis. In this review, we summarize the molecular basis for the development and progression of GBM as well as some emerging therapeutic approaches.

## 1. Introduction

Glioblastoma (GBM), often referred to as a grade IV astrocytoma, is an aggressive malignant tumor that occurs in the brain or spinal cord. While the incidence rate is relatively low (approximately 5 per 100,000 persons) when compared with other types of cancers [1], GBM is notorious for its poor prognosis with a median survival ranging from 12 to 18 months after the initial diagnosis [2]. Despite recent advances in diagnosis, surgical techniques, and therapeutic approaches, overall survival was not dramatically improved, with a one-year survival rate of 40.8% and a five-year survival rate of 6.8% [3]. Major challenges for GBM treatment are as follow. First, complete elimination of tumor tissue with surgery is very difficult due to its highly infiltrative nature [4]. Second, owing to the unique location of GBM, drugs should penetrate the blood brain barrier (BBB) to reach the tumor site. However, as a highly selective semipermeable barrier, the BBB prevents most of the clinically approved drugs entering the brain [5,6]. Third, despite aggressive resection and subsequent radiochemotherapy, almost all GBMs recur within the first year even at a distant cerebral locations [7]. Fourth, GBM actively operates several resistance mechanisms against conventional therapies, and subpopulation of tumor cells (named GBM stem cells) contribute to this drug resistance [8].

**Figure 1:**
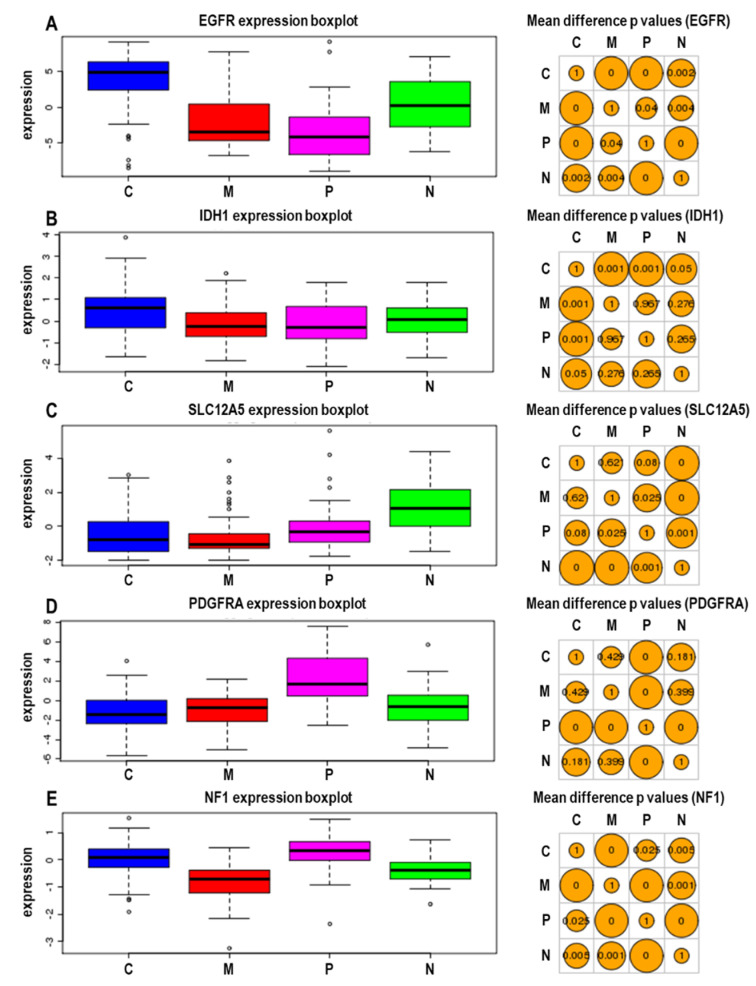
Relative gene expressions in four subtypes of GBM. Relative mRNA expressions (left) and mean difference p values between each subtype (right) of EGFR (**A**), IDH1 (**B**), SLC12A5 (**C**), PDGFRA (**D**), and NF1 (**E**). These data were derived from GBM-BioDP software [10]. C—classical; M—mesenchymal; P—proneural; N—neural.

Based on integrated multi-dimensional genomic analysis, GBMs can be classified into four subtypes: proneural, neural, classical, and mesenchymal (Figure 1). The proneural subtype features an abnormally high level of platelet-derived growth factor receptor A (PDGFRA) and mutations in the *IDH1* gene. The neural class exclusively expresses neuron markers such as *NEFL*, *GABRA1*, *SYT1,* and *SLC12A5*. The main feature of the classical subtype is a high-level *EGFR* gene amplification and the mesenchymal class presents a lower *NF1* expression. Furthermore, each subtype shows different clinical characteristics and treatment efficacy [9]. To overcome the current limitation on GBM treatment and develop ideal personalized therapies, improved molecular understanding of GBM pathogenesis is essential. In this review, we summarize the current knowledge of molecular pathogenesis and the current/emerging therapeutic approaches for GBM.

## 2. Molecular Pathogenesis of GBM

### 2.1. EGFR and EGFRvIII

The ErbB family of transmembrane proteins contains four receptor tyrosine kinases, the epidermal growth factor receptor EGFR (ERBB1), HER2 (ERBB2), HER3 (ERBB3), and HER4 (ERBB4) [11]. Of note, *EGFR* gene mutation, rearrangement, and amplification, as well as altered RNA splicing, were observed in 57.4% of primary GBM [12]. Although it was previously reported that HER2-specific T cells from GBM patients showed a potent anti-tumoral activity against HER2-positive GBM cells [13], the expression level of HER2 in GBM is still controversial. Whereas Liu et al. demonstrated that the HER2 mRNA expression was detected in 81.4% of GBM primary cells [14], other studies showed that the HER2 protein or the amplification of the *HER2* gene were absent or rarely detected in GBM [15,16]. Therefore, the contribution of HER2 to GBM tumorigenesis needs to be further determined.

**Figure 2:**
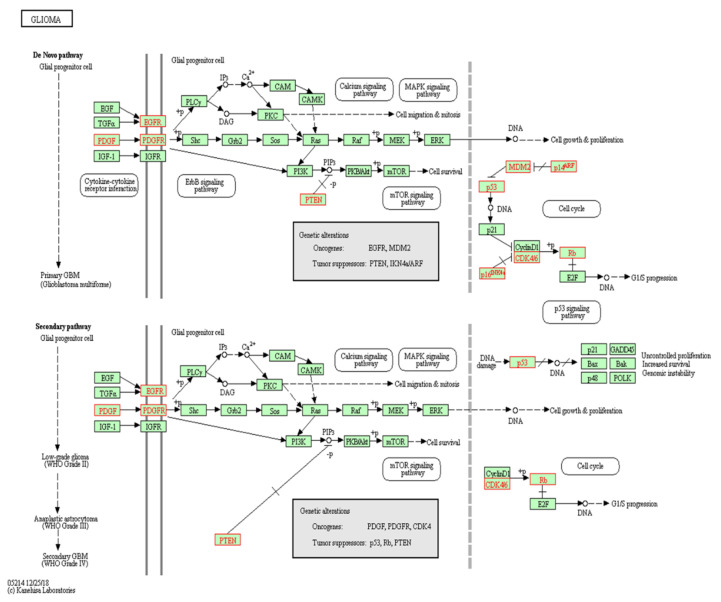
Genetic alteration pathways of GBM. Gene pathways related to pathogenesis in primary and secondary GBM (map05214). The data was derived from the Kyoto Encyclopedia of Gene and Genomes (KEGG) database [26,27,28].

EGFR plays fundamental roles in several physiological conditions such as the developmental process, differentiation, cell proliferation, and cell cycle control [17]. Upon activation, EGFR recruits the SH2-containing adaptor protein GRB2 in a preformed complex with SOS, which facilitates RAS activation (RAS-GTP) and the subsequent triggering of the RAF-MAPK/ERK kinase (MEK)-ERK1/2 signaling cascade [18,19]. Activated ERK proteins are then translocated into the nucleus where they invigorate specific transcription factors involved in cell proliferation [20]. Moreover, EGFR also activates Class I phosphoinositide-3 kinases (PI3K) by antagonizing the action of p85, a regulatory subunit of Class I PI3K [21]. The PI3K activation leads to the generation of PIP3, which in turn induces a conformational change in AKT. This conformational change allows PDK1 to activate AKT and the mammalian target of rapamycin complex 2 (mTORC2) [22]. This PI3K-PDK1-AKT-mTOR pathway contributes to cell growth, survival, and proliferation (Figure 2) [23]. Additionally, EGFR directly phosphorylates and stimulates STAT3 function in regulating cell stemness, migration, and transformation [24]. Active EGFR is also able to activate the PLC-PKC axis that is crucial for angiogenesis and cell proliferation, infiltration, and survival [25].

Amplification or activating mutations of *EGFR* lead to an abnormal upregulation of several downstream signaling pathways. In GBM, the most common oncogenic mutation of *EGFR*, termed *EGFRvIII*, is characterized by the deletion of exons 2 to 7, resulting in an in-frame loss of 267 amino acids in the extracellular domain of the EGFR protein [29]. The EGFR was the first receptor to be proposed as a target for cancer therapy. Indeed, phase I and II clinical studies showed that a peptide vaccine targeting EGFRvIII efficiently eliminated EGFRvIII-expressing GBM cells and extended both progression-free and overall patient survival [30]. However, unfortunately, a survival benefit was not confirmed in patients with newly diagnosed GBM from a recent randomized, double-blind, international phase III clinical trial [31], suggesting that therapeutic alternatives or new combinatorial approaches are still required.

### 2.2. IDH1/2 Mutation

Isocitrate dehydrogenase (IDH) enzymes, which contain three isoforms, play key roles in several major cellular metabolic processes, such as the tricarboxylic acid (TCA) cycle, glutamine metabolism, lipogenesis, and redox regulation [32]. Wild-type IDH1 and 2 localize in the cytoplasm and mitochondria, respectively, and catalyze the decarboxylation of isocitrate to alpha-ketoglutarate (α-KG) with reduction of NADP+ to NADPH [33]. NADPH is necessary to decrease the level of reactive oxygen species (ROS) in the cell and synthesize deoxynucleotides triphosphates (dNTPs) during DNA damage repair [34]. In contrast to the wild-type protein, the mutant IDH activity promotes NADPH oxidation to NADP+, which results in decreased levels of NADPH and increased ROS levels [35].

**Figure 3:**
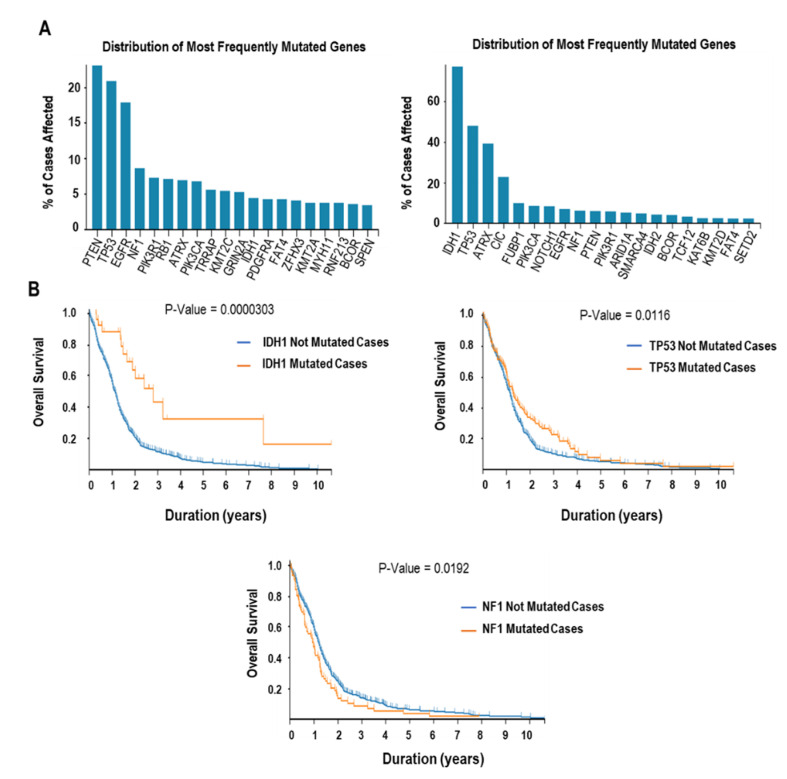
The frequency of mutated genes in GBM and their influence on overall survival. (**A**) The frequency of mutated genes in GBM (left) and low-grade gliomas (right). (**B**) Overall survival of GBM patients with (orange line) or without (blue line) mutation of IDH1, TP53, and NF1. These data were derived from Genomic Data Commons Data portal [37].

IDH1/2 mutations occur in 53% to 83% of grade II/III astrocytomas and oligodendrogliomas and in 54% of secondary glioblastoma, but only in 6.3% of primary glioblastomas (Figure 3A) [36].

It was shown that patients suffering from primary and secondary GBMs that contained IDH mutations had a significantly longer overall survival when compared with patients without these mutations (Figure 3B) [38]. Gain-of-function (GOF) mutations in *IDH1* (R132H) and *IDH2* (R172H) increase 2-hydroxyglutarate (2-HG) levels due to a defective function of αKG-dependent dioxygenases [39,40]. In fact, 2-HG acts as competitive inhibitor of αKG due to its high similarity to the binding position of αKG [41]. 2-HG also promotes the activity of the Egl nine homolog prolyl-hydroxylases by hypoxia inducible factors [42]. Similarly, increased 2-HG levels caused by *IDH1* mutations activate HIF1α-inducible genes, such as the vascular endothelial growth factor (VEGF) and GLUT-1 [43,44]. *IDH1/2* mutations were discovered to occur concomitantly to mutations in *TP53*, *ATRX*, and *TERT* promoter, to codeletions of 1p/19q, and to molecular alterations in PTEN and EGFR [45,46]. Furthermore, IDH1 mutations decrease the expression of the astrocyte marker GFAP and increase the expression of the neural stem cell (NSC) marker NESTIN [47]. *IDH* mutations result in hypermethylation of a subset of CTCF (CCCTC-binding factor) binding sites leading to a significant alteration in the three-dimensional structure of DNA [48]. This GOF mutant IDH also enhances PDGFRA expression, especially in CpG islands [49].

Based on the aforementioned prognostic role of IDH mutations, the World Health Organization (WHO) recently suggested new classification of GBM into (1) IDH-wildtype or IDH negative GBM (about 90 % of cases) that preferentially arises in patients over 55 years of age and shows mostly poor prognosis; (2) IDH-mutant or IDH positive GBM (about 10% of cases) that predominates in younger patients and shows better prognosis [50,51].

### 2.3. TP53 Mutation

As the “guardian of the genome” and “cellular gatekeeper”, the tumor suppressor *TP53* modulates anti-proliferative cellular responses by regulating key effector genes [52,53,54]. Upon a cellular stress, such as replicative or oxidative stress, the p53 protein is stabilized and accumulated inside the cells. Upon DNA damage, the ATM/ATR pathway-induced p53 activation leads to the G1 arrest mainly through the transactivation of *p21^Waf1/Cip1^* [55]. In addition, p53 was also shown to result in the G2/M arrest through disturbing the cyclin B1/CDC2 complex in response to DNA damage [56]. The p53-p21 axis is also critical for the cell senescence program. In fact, when p14ARF detects senescence signals, it binds to MDM2 and blocks the MDM2-dependent degradation of p53. In turn, the upregulation of p21^Waf1/Cip1^ leads to dephosphorylation and activation of the RB protein, which subsequently suppresses E2F, a potent inducer of cell proliferation [57]. In animal models, it was already reported that the reactivation of p53 results in complete tumor regression, primarily through the induction of the cellular senescence program [58]. p53 is highly implicated in the apoptosis program in both a transcription-dependent and -independent manner. Under apoptotic stimuli, p53 transcriptionally regulates a subset of genes involved in apoptosis, including *BAX*, *FAS*, *BBC3* (also known as *PUMA*), and *BIRC5* (encoding SURVIVIN), to coordinate the apoptotic program [59]. Moreover, p53 has extranuclear activities during apoptosis. For example, in response to various cell death-inducing signals, p53 is rapidly relocated to the mitochondria and associates with the outer mitochondrial membrane [60]. Once it is relocated, p53 facilitates mitochondrial outer membrane permeabilization, thereby liberating pro-apoptotic factors from the mitochondrial intermembrane space [61]. Additionally, p53 also controls autophagy by transcriptionally upregulating *DRAM* (damage-regulated autophagy modulator), an effector of macroautophagy and an essential factor for p53-mediated cell death [62].

Mutation or deletion of *TP53* was observed in 27.9% of primary GBM (Figure 3A) [12]. When combined with other genetic factors that affect the abundance or activity of the p53 protein, including amplification of *MDM* genes and/or deletion of *CDKN2A*, the p53 pathway is considered dysregulated in 85.3% of GBMs. Previous studies already showed that the malignant progression of astrocytoma toward end-stage GBM is accompanied by sequential genetic and epigenetic alterations including the amplification/mutation of the *EGFR* gene and the hypermethylation of the chromosomal region around the *TP53* gene [63]. Restoration of p53 function in GBM cells was shown to secrete inhibitors of capillary endothelial cell migration, suggesting that p53 has an anti-angiogenic role in GBM. Interestingly, however, recent findings have demonstrated that the p53 GOF mutant may possess neomorphic roles, contrary to wild-type p53. GOF mutations in *TP53* contribute to tumor malignancies in GBM by promoting cell proliferation [64], neo-angiogenesis [65], and aberrant activation of inflammatory responses [66].

### 2.4. PTEN Mutation

The tumor suppressor PTEN has an important role as a lipid and protein phosphatase, regulating cell proliferation, adhesion, and invasion, and DNA damage repair [67]. PTEN dephosphorylates the PIP3 to create PIP2, and thereby inhibits the PI3K/AKT pathway [68,69]. The PI3K/AKT pathway is dysregulated in GBM and supports cell proliferation by promoting the presence of anti-apoptotic signals in the cell [70]. PTEN induces high level of H3F3B (H3.3) by inhibiting DAXX (death domain-associated protein) and thus suppresses the expression of oncogenic genes [71]. The PTEN nuclear localization regulates the tumor suppressor TP53 by a PI3K/AKT-independent mechanism, which leads to the control of cyclin D1 expression and to an increase in the ubiquitin-dependent degradation of tumor-promoting proteins, such as PLK1 and AURK [72,73]. In contrast, the absence of nuclear PTEN is associated with the overexpression of FBXO22 and with proliferation in various cancer types [74].

The loss of PTEN or mutations in the *PTEN* gene are observed in 5% to 40% of GBMs and were shown to affect the cell size or number (Figure 3A) [75]. The PTEN mutant leads to upregulation of ARL4C, which is related to filopodium formation that in turn improves metastasis abilities of GBM cells [76]. Chen et al. found that deficiency or mutation of PTEN increased lysyl oxidase (LOX) expression to support macrophage infiltration via SRC/AKT-YAP1 pathway, which leads to the progression and survival of GBM [77]. Additionally, PTEN loss results in the blockage of T-cell infiltration and autophagy activation through the induction of expression of immunosuppressive cytokines, such as IL10, TGFβ, and PGE2 [78]. PTEN also plays a role in inflammatory responses, where its loss or mutation lead to upregulation of IL2 and STAT5 phosphorylation and to downregulation of STAT3 phosphorylation [79].

### 2.5. NF1 Mutation

The *NF1* gene, located on chromosome 17, functions as a key negative regulator of the RAS pathway. Genetic alterations on the *NF1* gene have often been reported in numerous cancer types [80], and mutation or deletion of *NF1* was observed in 10% of GBM cases (Figure 3A) [12]. The *NF1* gene encodes neurofibromin, a functional GTPase-activating protein (GAP) that downregulates RAS (including HRAS, NRAS, and KRAS) activity by facilitating the hydrolysis of RAS-GTP [81]. Abnormal activation of RAS catalyzes the epithelial–mesenchymal transition (EMT) by upregulating EMT-linked transcription factors including SNAIL, SLUG, TWIST, ZEB1, and ZEB2 [82].

Although *NF1* was known as the third most prevalent disrupted gene in GBM [83], the role of the NF1 protein in gliomagenesis was not yet well defined. Of note, *NF1* loss or mutations, including splice site mutations, missense mutations, nonsense mutations, and frameshift indels, have been more frequently found in aggressive mesenchymal GBM than in other subtypes [84]. Llaguno et al. showed that loss of *NF1* in lineage-restricted central nervous system progenitor cells could induce malignant transformation [85]. NF1 was also reported to suppress glioma metastasis and invasion through its leucine-rich domain [86]. More recently, Wang et al. demonstrated that NF1 inactivation induces the infiltration of tumor-associated macrophages/microglia, suggesting a new role for NF1 in tumor microenvironment regulation [87].

### 2.6. TERT Promoter Mutation

To maintain chromosomal integrity and genome stability, eukaryotic cells have the protective nucleoprotein complexes, called telomeres, at chromosomal ends. Telomeric DNA contains double-stranded hexameric repeat sequences ending in terminal 3′ G-rich single-stranded overhangs [88]. Telomeres shortening occurs in each round of cell division and limits the cell division by inducing replicative senescence or apoptosis [89]. Telomerase is a specialized DNA polymerase with reverse transcriptase activity, capable of maintaining the length of telomeres and promoting cellular immortalization. The telomerase complex consists of two subunits: a telomerase RNA component (TERC) and a functional catalytic protein subunit called TERT (telomerase reverse transcriptase) [90]. TERC is ubiquitously expressed in most cell types and even in telomerase-deficient cells, and it serves as a template for telomere extension [91]. In contrast, TERT expression is highly regulated, which is absent or present in low levels in somatic cells but upregulated in several types of cancer cells [92]. Telomerase inhibition with short telomeres has been regarded as a potent barrier to oncogenesis [93].

Cancer cells often overcome cellular senescence by telomerase activation. Furthermore, in cancer cells, TERT upregulation can be achieved by TERT amplifications, TERT promoter mutations, and TERT promoter methylation [94,95]. In gliomas, the prevalence of mutations on the TERT promoter is remarkably high in primary GBMs (approximately 80%) (Figure 3A) [96], and recent evidence suggested the presence of TERT promoter mutations as an unfavorable prognostic factor in GBM [97]. Recurrent non-coding point mutations within the TERT promoter are predominantly observed at two nucleotide positions: −124 and −146 bp upstream from the ATG, denoted C228T and C250T, respectively [98].

## 3. Current Therapies for Glioblastoma

### 3.1. Surgery

The standard treatment for GBMs is maximal surgical resection, followed by radiation and chemotherapy [99]. The primary objective of surgery is to obtain tissue specimen for pathological diagnosis and eliminate as much of the tumor as possible without damaging the surrounding normal brain tissue. In addition, surgical treatment can improve conditions for following therapeutic approaches [100].

For operative candidate selection, medical conditions of the patient should be taken into account. Given that age has been regarded as the most essential prognostic factor, ages of patients need to be considered for selection of therapeutic options. Surgery may cause more side effects in aged patients and the efficacy of surgery in the elderly patients still remains controversial [101]. However, considering that the elderly may receive less intensive radio- and chemo-therapies, elderly patients with GBM seemed to profit from surgery as younger patients. Tumor size and location also needs to be considered. Due to the infiltrative feature of glioma, GBMs are frequently found near or within eloquent cortex [102]. A recent study demonstrated that malignancies residing in an eloquent area are major hindrances to surgical resection due to the high risk of postoperative neurological deficits [103]. Indeed, it was previously reported that postoperative neurological deficits did not occur in patients when the resection margins were over 2 cm of the eloquent cortex [104]. However, lower resection margins led to postoperative neurological impairments more frequently.

The most important predictor of postoperative outcome is the extent of resection (EOR). Lacroix et al. analyzed the balance between EOR and survival advantage and found that significantly improved survival was associated with an EOR of 98% or more [105]. While the median survival was 13 months for an EOR of 98% or more, survival disbenefit at an EOR of less than 98% was 4.2 months. Another following study also confirmed the positive correlation between EOR and median overall survival [106].

### 3.2. Radiation

After surgical management, when the wound is healed, concurrent radiochemotherapy is applied. The primary objective of radiotherapy is the clearance of residual tumor cells that have infiltrated the surrounding noncancerous tissue. In the standard course of radiation therapy, multiple irradiations of defined dose called “fractions” are administrated to the tumor site and margin. As a result of the phase III trial, the median survival for patients who received radiotherapy (focal radiation in daily fractions of 1.8 Gy given 5 days per week, for a total dose of 50 Gy) was 29.1 weeks, as compared with 16.9 weeks for the patients who did not take radiotherapy [107]. Considering the adverse effect of radiotherapy, shorter course of irradiation (40 Gy in 15 fractions over 3 weeks), also called hypofractionation, were clinically tested especially in the elderly patients. The result showed that there was no difference in survival between 6-week and 3-week courses, suggesting that hypofractionated radiotherapy could be a reasonable treatment approach for the aged patients [108].

### 3.3. Temozolomide

After surgical resection, concomitant treatment with radiotherapy plus temozolomide is internationally approved, first-line standard approach for GBM. Compared to radiotherapy alone, additional usage of temozolomide provided 2.5 months of the median survival benefit and increased the two-year survival rate from 10.4% to 26.5% [109]. Temozolomide is an orally administered alkylating agent that induces base mismatches followed by the DNA double strand breakage, eventually leading to cell death [110]. Recently, temozolomide was additionally known to promote cellular senescence and inhibit DNA repair system in GBM cells [111]. Notably, temozolomide can penetrate the BBB and reaches the tumor site with approximately 20% of brain to plasma AUC ratio [112]. Without hepatic metabolic activation, temozolomide is spontaneously hydrolyzed at physiologic pH to active form [113].

Although temozolomide significantly improved overall survival, it also suffers from some limitations. Due to its short half-life of 1.9 h in plasma [114], prolonged systemic administration at high dose is required, but side effects follow. High concentration of temozolomide is toxic, particularly to hematopoietic cells, resulting in several hematologic events including neutropenia and thrombocytopenia [115]. To remedy this drawback, nanocarriers and temozolomide-conjugated compounds are now actively developed [116]. These approaches may reduce the administrative dose of temozolomide by enhancing brain permeability and/or increasing the stability in plasma and/or upregulating the binding affinity to receptors expressed in GBM. For example, Fang et al. devised a chitosan-based nanoparticle to improve the therapeutic efficacy of temozolomide. When bound to this nanoparticle, temozolomide exhibited sustained stability at physiological pH by almost seven-fold greater than unbound temozolomide. The authors also utilized chlorotoxin as a supportive platform to upgrade target specificity against GBM and inhibit tumor invasion. This nanoparticle conjugated with temozolomide and chlorotoxin was proven to penetrate an intact BBB in mice model, showing great promise for advances in the treatment of GBM [117].

**Figure 4:**
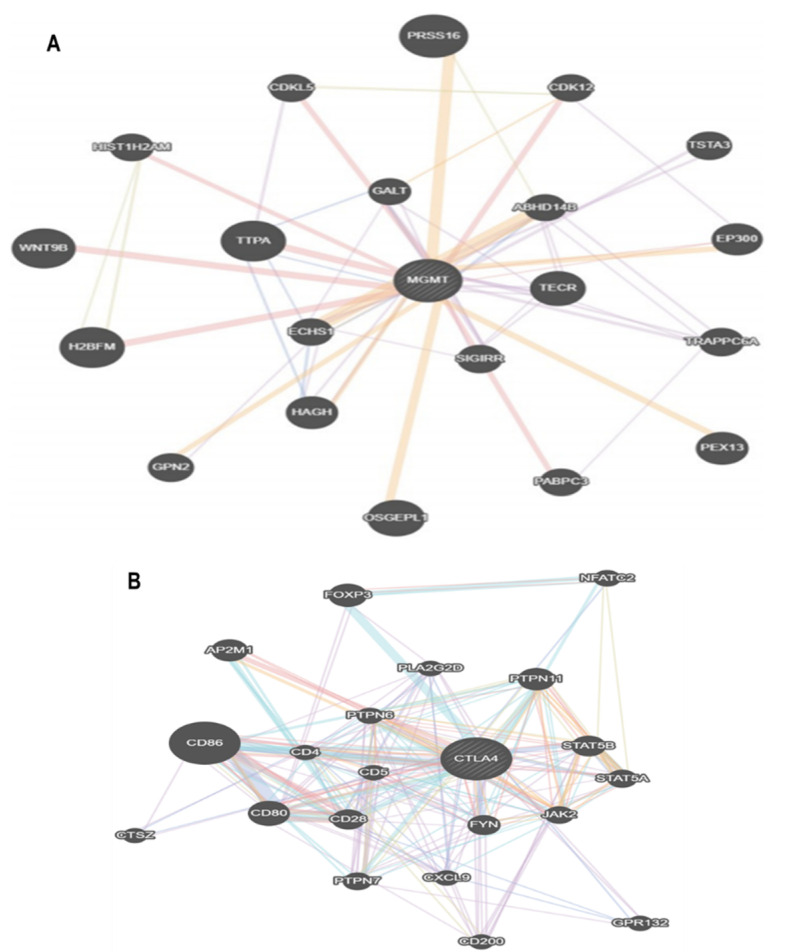
Genetic network showing the MGMT and CTLA4. (**A**) The gene-gene interaction network of MGMT related to GBM treatment. (**B**) The gene-gene interaction network of CTLA4. These data were derived from GeneMANIA database [121].

Another important issue regarding temozolomide is drug resistance. The most studied main culprit responsible for the resistance to temozolomide is O6-methylguanine-DNA methyl transferase (MGMT). MGMT is a DNA repair protein, which rapidly reverses DNA lesion O6-methylguanine to guanine, thereby suppressing the lethal cross-linking [118]. Therefore, the expression level of MGMT has been a reasonable predictor of the resistance of tumors to alkylating agents. Additionally, in glioma cells, tumor cells with low MGMT activity showed higher sensitivity to temozolomide and reduced clonogenic survival after exposure to temozolomide [119,120]. While the *MGMT* gene mutation was not frequently reported, MGMT expression can be regulated in various ways (Figure 4A).

At DNA level, CpG methylation within the *MGMT* promoter contributes to the gene expression modulation. Hegi et al. reported that *MGMT* promoter methylation was observed in approximately 50% of GBM, and suggested the hypermethylation on the *MGMT* promoter as treatment-independent favorable prognostic factor [122]. Consistently, Esteller et al. reported that *MGMT* promoter hypermethylation increased overall survival and the time to progression of disease [118]. In addition to promoter methylation, histone modification status and distal enhancer also control transcription of the *MGMT* gene. Recently, Chen et al. identified that the enhancer, located at 560 kb away from the *MGMT* promoter, positively regulates the transcription of MGMT even when the promoter is highly methylated. The enhancer activity is typically regulated by histone modifications of surrounding nucleosomes, and indeed, H3K4me1 and H3K27ac that mark active enhancers are enriched at the enhancer of *MGMT* in temozolomide-resistant GBM cells in which MGMT is expressed abundantly [123]. On the contrary, however, emerging evidence suggests that MGMT expression level does not solely reflect the temozolomide resistance. Despite a deficiency of MGMT expression, temozolomide shows little or no effect in some GBM cases [124]. In pediatric GBM, the ATM-mediated base excision repair pathway confers therapeutic resistance to temozolomide, irrelevant to MGMT expression [125]. In addition, HOXA9/HOXA10 expression regulated by PI3K contributes to temozolomide resistance independently of MGMT level, leading to shorter survival rate [126].

Autophagy is also relevant in GBM responsiveness to temozolomide. Autophagy is a self-degradative process where unnecessary or dysfunctional sub-cellular organelles are engulfed by autophagosomes, followed by the fusion with lysosomes [127]. Autophagy shows critical roles in the maintenance of cell homeostasis and genomic integrity, and it also has a protective function on cells against stress [128]. Previously, it was shown that temozolomide induces autophagy and pharmacological inhibition of autophagy enhances the anti-tumor activity of temozolomide [129]. However, interestingly, enforced upregulation of autophagy can also suppress the development of GBM. Recently, EMC6 was identified as novel positive regulator of autophagy, and GBM cell showed higher sensitivity to temozolomide and reduced growth potential when EMC6 is overexpressed [130].

### 3.4. Bevacizumab

Owing to the infiltrative nature of GBM, examination of the surrounding GBM commonly identifies the existence of infiltrative tumor cells [131]. Given that infiltration and growth of tumors are facilitated by angiogenesis, the communication between cancer cells and blood vessels is important [132]. Surprisingly, it was previously reported that GBM stem cells have a potential of transdifferentiating into endothelial cells or pericytes to support new blood vessel generation, highlighting the significance of angiogenesis [133,134].

Tumor angiogenesis is modulated by several diffusible factors that are produced and released by tumor cells [135]. Inter alia, vascular endothelial growth factor (VEGF) plays as a key angiogenic inducer, and it has been implicated in tumor pathogenesis and angiogenesis [136]. In this context, about 50 years ago, Folkman already proposed that inhibition of angiogenesis could be an effective therapeutic approach for cancer treatment, and clinical trials with anti-VEGF antibodies and other VEGF inhibitors have been performed [132]. As expected, therapeutic exploitation targeting VEGF or VEGF receptor showed substantial clinical improvement in several types of malignancies [137].

Bevacizumab is a humanized anti-VEGF monoclonal antibody, and it was first approved for anti-angiogenic therapy for the management of advanced colon cancer in 2004. Bevacizumab was additionally granted FDA approval in multiple cancers including non-small cell lung cancer, metastatic breast cancer, metastatic renal cell carcinoma, and GBM [138]. For GBM treatment, however, while bevacizumab has been shown to provide a progression-free survival benefit in patients with newly diagnosed or recurrent GBM [131,139,140], the overall survival was not highly improved with bevacizumab. Currently, combination therapy of bevacizumab with other drugs are actively under evaluation. Although phase I/II study of bevacizumab with BKM120, an oral PI3K inhibitor, was performed, the efficacy of this regimen was unsatisfactory [141]. Phase II trial of bevacizumab with dasatinib, an ATP-competitive tyrosine kinase inhibitor, also failed to show significantly improved outcome [142]. Recently, Hata et al. showed a promising result that the overall survival of GBM patients was significantly enhanced when bevacizumab was utilized complementary to temozolomide [143].

### 3.5. Immunotherapy

Targeting checkpoints of immune cell activation has shown recent success in the treatment against several types of malignancies [144]. An immune checkpoint has critical roles to maintain immune homeostasis and prevent abnormal activation/autoimmunity. However, some cancer cells maliciously stimulate immune checkpoints, leading to the blockade of anti-tumor T cell responses [145]. Therefore, immune checkpoint inhibitors remove inhibitory signals of T-cell activation, and in turn, invigorate the immune system of cancer patients to extinct tumor cells. So far, two inhibitory checkpoints, cytotoxic T-lymphocyte-associated protein 4 (CTLA-4) and programmed cell death protein 1 (PD-1), are the most promising targets. To date, six drugs blocking the PD-1/PD-L1 pathway and one drug suppressing the CTLA-4 pathway were already approved by the U.S. Food and Drug Administration (FDA) for the treatment of specific cancers (Figure 4B) [144].

Similar to other types of cancers, GBM was reported to disturb the immune system. Notably, the expression of PD-L1 was shown to be highly upregulated in GBM after the loss of PTEN and activation of PI3K signaling [146]. Nonetheless, targeting the PD-1/PD-L1 pathway was not effective for GBM clearance, most likely due to a low immunogenic response of GBM and immunosuppressive microenvironment surrounding tumor [147]. Indeed, as a result of phase III clinical trial, nivolumab, PD-1 inhibitor, did not show survival benefits [148]. The combination of nivolumab with ipilimumab, a monoclonal antibody targeting CTLA-4, also failed to improve efficacy outcomes [149]. To make a breakthrough, several clinical trials are in progress to evaluate combination therapy of PD-1/PD-L1 inhibitor with radiotherapy or antibodies against CTLA-4, TIM-3 LAG-3, IDO, or OX-40.

## 4. New Drug Candidates against Glioblastoma

**Figure 5:**
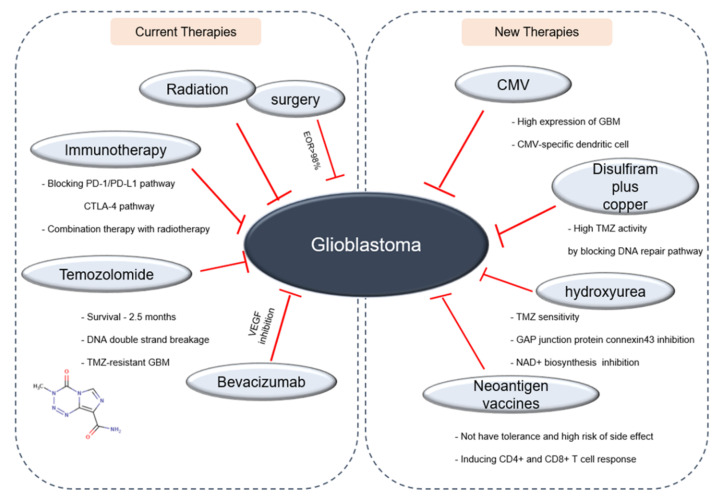
A schematic diagram showing the GBM therapies. A diagram demonstrates the current and new promising therapies.

Novel immunotherapies for GBM are now ongoing. Previously, *Cytomegalovirus* (CMV) has been reported to have active roles in the pathogenesis of several tumors, and interestingly, CMV protein pp65 was proven to be present in a high percentage of GBM but not in surrounding normal brain (Figure 5) [150]. Based on these reports, clinical trials investigating the use of CMV-specific dendritic cell (DC) have been progressively conducted. Prins et al. reported a patient case where extremely robust CD8+ T-cell response to the pp65 was developed immediately after a single injection of autologous tumor lysate-pulsed DC [151]. The following trial demonstrated that pre-conditioning DCs with tetanus/diphtheria toxoid significantly enhanced DC migration to draining lymph nodes and eventually provided survival benefit [152,153]. Reproducibility in these clinical data was confirmed by long term follow-up with larger sample size [154].

Strategies to overcome the drug resistance are now actively considered. Lun et al. found that disulfiram plus copper supplement showed the cytotoxicity against patient-derived tumor-initiating cells that confer resistance to DNA-damaging therapies (temozolomide and radiation). Disulfiram plus copper upregulated temozolomide activity by attenuating DNA repair pathways in vitro. Disulfiram plus copper, also in vivo, suppressed tumor growth and prolonged survival in newly diagnosed, recurrent, and temozolomide-resistant GBM intracranial mice models [155]. More recently, Teng et al. found that hydroxyurea augmented the response of GBM cells and patient-derived stemlike cells against temozolomide. The FDA-approved drug hydroxyurea made newly diagnosed and recurrent GBM to sensitize to temozolomide, and provided survival benefit [156]. In addition, the inhibition of the gap junction protein connexin 43 [157] or dual inhibition of NAD+ biosynthesis and base excision repair [158] has also been considered to overcome temozolomide resistance.

Currently, personalized neoantigen vaccines are actively developed. Neoantigens are derived from tumor-specific somatic mutations that are not identified in normal human genome. Because neoantigens are specifically expressed in tumor tissues and they are specific to each individual patient or tumor, they are not subject to central tolerance and have a low risk of side effects [159]. Given that neoantigen vaccines show a potent anti-tumor activity by inducing both CD4+ and CD8+ T cell responses [160], clinical trials utilizing neoantigen vaccines have been conducted [161]. Recent evidence suggests that neoantigen vaccines showed clinical benefit for patients with GBM [162,163]. Although further optimization and clinical studies are required, neoantigen vaccines represent a new promising approach of precision medicine [164].

## 5. Future Directions

GBM is still thought of as an incurable disease. The first-line standard treatments for GBM has failed to provide dramatic survival benefit, and immunotherapies, holding great promise for several types of cancers, have not come up to expectation yet in GBM clearance. However, many researchers did not surrender to aggressive brain tumor, and scientific findings on GBM have been accumulated. Genomic profiling of GBM tissues has disclosed new potential molecular targets that are abnormally expressed in tumors, and development of novel immunotherapies, neoantigen vaccine, and strategies to overcome the drug resistance is now ongoing. These efforts will eventually surmount the current difficulties on GBM treatment and make ideal personalized therapies possible.

## References

[1] Tamimi A.F., Juweid M., De Vleeschouwer S. (2017). Epidemiology and outcome of glioblastoma. Glioblastoma.

[2] Caruso R., Pesce A., Wierzbicki V. (2017). A very rare case report of long-term survival: A patient operated on in 1994 of glioblastoma multiforme and currently in perfect health. Int. J. Surg. Case Rep..

[3] Ostrom Q.T., Cioffi G., Gittleman H., Patil N., Waite K., Kruchko C., Barnholtz-Sloan J.S. (2019). CBTRUS Statistical Report: Primary Brain and Other Central Nervous System Tumors Diagnosed in the United States in 2012–2016. Neuro-Oncology.

[4] Wen P.Y., Kesari S. (2008). Malignant gliomas in adults. N. Engl. J. Med..

[5] Xu Y.Y., Gao P., Sun Y., Duan Y.R. (2015). Development of targeted therapies in treatment of glioblastoma. Cancer Biol. Med..

[6] Taylor O.G., Brzozowski J.S., Skelding K.A. (2019). Glioblastoma Multiforme: An Overview of Emerging Therapeutic Targets. Front. Oncol..

[7] Ito H., Nakashima H., Chiocca E.A. (2019). Molecular responses to immune checkpoint blockade in glioblastoma. Nat. Med..

[8] Jiapaer S., Furuta T., Tanaka S., Kitabayashi T., Nakada M. (2018). Potential Strategies Overcoming the Temozolomide Resistance for Glioblastoma. Neurol. Med. Chir..

[9] Verhaak R.G., Hoadley K.A., Purdom E., Wang V., Qi Y., Wilkerson M.D., Miller C.R., Ding L., Golub T., Mesirov J.P. (2010). Integrated genomic analysis identifies clinically relevant subtypes of glioblastoma characterized by abnormalities in PDGFRA, IDH1, EGFR, and NF1. Cancer Cell.

[10] Celiku O., Johnson S., Zhao S., Camphausen K., Shankavaram U. (2014). Visualizing molecular profiles of glioblastoma with GBM-BioDP. PLoS ONE.

[11] Arteaga C.L., Engelman J.A. (2014). ERBB receptors: From oncogene discovery to basic science to mechanism-based cancer therapeutics. Cancer Cell.

[12] Brennan C.W., Verhaak R.G., McKenna A., Campos B., Noushmehr H., Salama S.R., Zheng S., Chakravarty D., Sanborn J.Z., Berman S.H. (2013). The somatic genomic landscape of glioblastoma. Cell.

[13] Ahmed N., Salsman V.S., Kew Y., Shaffer D., Powell S., Zhang Y.J., Grossman R.G., Heslop H.E., Gottschalk S. (2010). HER2-specific T cells target primary glioblastoma stem cells and induce regression of autologous experimental tumors. Clin. Cancer Res..

[14] Liu G., Ying H., Zeng G., Wheeler C.J., Black K.L., Yu J.S. (2004). HER-2, gp100, and MAGE-1 are expressed in human glioblastoma and recognized by cytotoxic T cells. Cancer Res..

[15] Haynik D.M., Roma A.A., Prayson R.A. (2007). HER-2/neu expression in glioblastoma multiforme. Appl. Immunohistochem. Mol. Morphol..

[16] Koka V., Potti A., Forseen S.E., Pervez H., Fraiman G.N., Koch M., Levitt R. (2003). Role of Her-2/neu overexpression and clinical determinants of early mortality in glioblastoma multiforme. Am. J. Clin. Oncol..

[17] Citri A., Yarden Y. (2006). EGF-ERBB signalling: Towards the systems level. Nat. Rev. Mol. Cell Biol..

[18] Lowenstein E.J., Daly R.J., Batzer A.G., Li W., Margolis B., Lammers R., Ullrich A., Skolnik E.Y., Bar-Sagi D., Schlessinger J. (1992). The SH2 and SH3 domain-containing protein GRB2 links receptor tyrosine kinases to ras signaling. Cell.

[19] Scaltriti M., Baselga J. (2006). The epidermal growth factor receptor pathway: A model for targeted therapy. Clin. Cancer Res..

[20] Hill C.S., Treisman R. (1995). Transcriptional regulation by extracellular signals: Mechanisms and specificity. Cell.

[21] Mattoon D.R., Lamothe B., Lax I., Schlessinger J. (2004). The docking protein Gab1 is the primary mediator of EGF-stimulated activation of the PI-3K/Akt cell survival pathway. BMC Biol..

[22] Klempner S.J., Myers A.P., Cantley L.C. (2013). What a tangled web we weave: Emerging resistance mechanisms to inhibition of the phosphoinositide 3-kinase pathway. Cancer Discov..

[23] Bunney T.D., Katan M. (2010). Phosphoinositide signalling in cancer: Beyond PI3K and PTEN. Nat. Rev. Cancer.

[24] Park O.K., Schaefer T.S., Nathans D. (1996). In vitro activation of Stat3 by epidermal growth factor receptor kinase. Proc. Natl. Acad. Sci. USA.

[25] Kadamur G., Ross E.M. (2013). Mammalian phospholipase C. Ann. Rev. Physiol..

[26] Kanehisa M., Goto S. (2000). KEGG: Kyoto encyclopedia of genes and genomes. Nucleic Acids Res..

[27] Kanehisa M., Sato Y., Furumichi M., Morishima K., Tanabe M. (2019). New approach for understanding genome variations in KEGG. Nucleic Acids Res..

[28] Kanehisa M. (2019). Toward understanding the origin and evolution of cellular organisms. Protein Sci..

[29] Gan H.K., Kaye A.H., Luwor R.B. (2009). The EGFRvIII variant in glioblastoma multiforme. J. Clin. Neurosci..

[30] Sampson J.H., Aldape K.D., Archer G.E., Coan A., Desjardins A., Friedman A.H., Friedman H.S., Gilbert M.R., Herndon J.E., McLendon R.E. (2011). Greater chemotherapy-induced lymphopenia enhances tumor-specific immune responses that eliminate EGFRvIII-expressing tumor cells in patients with glioblastoma. Neuro-Oncology.

[31] Weller M., Butowski N., Tran D.D., Recht L.D., Lim M., Hirte H., Ashby L., Mechtler L., Goldlust S.A., Iwamoto F. (2017). Rindopepimut with temozolomide for patients with newly diagnosed, EGFRvIII-expressing glioblastoma (ACT IV): A randomised, double-blind, international phase 3 trial. Lancet Oncol..

[32] Han S., Liu Y., Cai S.J., Qian M., Ding J., Larion M., Gilbert M.R., Yang C. (2020). IDH mutation in glioma: Molecular mechanisms and potential therapeutic targets. Br. J. Cancer.

[33] Dang L., White D.W., Gross S., Bennett B.D., Bittinger M.A., Driggers E.M., Fantin V.R., Jang H.G., Jin S., Keenan M.C. (2009). Cancer-associated IDH1 mutations produce 2-hydroxyglutarate. Nature.

[34] Molenaar R.J., Maciejewski J.P., Wilmink J.W., van Noorden C.J.F. (2018). Wild-type and mutated IDH1/2 enzymes and therapy responses. Oncogene.

[35] Fack F., Tardito S., Hochart G., Oudin A., Zheng L., Fritah S., Golebiewska A., Nazarov P.V., Bernard A., Hau A.C. (2017). Altered metabolic landscape in IDH-mutant gliomas affects phospholipid, energy, and oxidative stress pathways. EMBO Mol. Med..

[36] Arita H., Narita Y., Yoshida A., Hashimoto N., Yoshimine T., Ichimura K. (2015). IDH1/2 mutation detection in gliomas. Brain Tumor Pathol..

[37] Grossman R.L., Heath A.P., Ferretti V., Varmus H.E., Lowy D.R., Kibbe W.A., Staudt L.M. (2016). Toward a Shared Vision for Cancer Genomic Data. N. Engl. J. Med..

[38] Khan I., Waqas M., Shamim M.S. (2017). Prognostic significance of IDH 1 mutation in patients with glioblastoma multiforme. J. Pak. Med. Assoc..

[39] Zhang C., Moore L.M., Li X., Yung W.K., Zhang W. (2013). IDH1/2 mutations target a key hallmark of cancer by deregulating cellular metabolism in glioma. Neuro-Oncology.

[40] Parsons D.W., Jones S., Zhang X., Lin J.C., Leary R.J., Angenendt P., Mankoo P., Carter H., Siu I.M., Gallia G.L. (2008). An integrated genomic analysis of human glioblastoma multiforme. Science.

[41] Teng X., Emmett M.J., Lazar M.A., Goldberg E., Rabinowitz J.D. (2016). Lactate Dehydrogenase C Produces S-2-Hydroxyglutarate in Mouse Testis. ACS Chem. Biol..

[42] Miller J.J., Shih H.A., Andronesi O.C., Cahill D.P. (2017). Isocitrate dehydrogenase-mutant glioma: Evolving clinical and therapeutic implications. Cancer.

[43] Yalaza C., Ak H., Cagli M.S., Ozgiray E., Atay S., Aydin H.H. (2017). R132H Mutation in IDH1 Gene is Associated with Increased Tumor HIF1-Alpha and Serum VEGF Levels in Primary Glioblastoma Multiforme. Ann. Clin. Lab. Sci..

[44] Lu J., Xu L., Zou Y., Yang R.X., Fan Y., Zhang W., Yu D., Yao Y.G. (2014). IDH1 p.R132 mutations may not be actively involved in the carcinogenesis of hepatocellular carcinoma. Med. Sci. Monit..

[45] Dang L., Yen K., Attar E.C. (2016). IDH mutations in cancer and progress toward development of targeted therapeutics. Ann. Oncol..

[46] Mondesir J., Willekens C., Touat M., de Botton S. (2016). IDH1 and IDH2 mutations as novel therapeutic targets: Current perspectives. J. Blood Med..

[47] Lu C., Ward P.S., Kapoor G.S., Rohle D., Turcan S., Abdel-Wahab O., Edwards C.R., Khanin R., Figueroa M.E., Melnick A. (2012). IDH mutation impairs histone demethylation and results in a block to cell differentiation. Nature.

[48] Starke R.M., Connolly E.S., Komotar R.J. (2016). Isocitrate Dehydrogenase Mutation Leads to Alteration in 3-Dimensional DNA Structure and Oncogene Activation in Gliomas. Neurosurgery.

[49] Flavahan W.A., Drier Y., Liau B.B., Gillespie S.M., Venteicher A.S., Stemmer-Rachamimov A.O., Suva M.L., Bernstein B.E. (2016). Insulator dysfunction and oncogene activation in IDH mutant gliomas. Nature.

[50] Ohgaki H., Kleihues P. (2013). The definition of primary and secondary glioblastoma. Clin. Cancer Res..

[51] Louis D.N., Perry A., Reifenberger G., von Deimling A., Figarella-Branger D., Cavenee W.K., Ohgaki H., Wiestler O.D., Kleihues P., Ellison D.W. (2016). The 2016 World Health Organization Classification of Tumors of the Central Nervous System: A summary. Acta Neuropathol..

[52] Zilfou J.T., Lowe S.W. (2009). Tumor suppressive functions of p53. Cold Spring Harb. Perspect. Biol..

[53] Lane D.P. (1992). Cancer. p53, guardian of the genome. Nature.

[54] Levine A.J. (1997). p53, the cellular gatekeeper for growth and division. Cell.

[55] Deng C., Zhang P., Harper J.W., Elledge S.J., Leder P. (1995). Mice lacking p21CIP1/WAF1 undergo normal development, but are defective in G1 checkpoint control. Cell.

[56] Hermeking H., Lengauer C., Polyak K., He T.C., Zhang L., Thiagalingam S., Kinzler K.W., Vogelstein B. (1997). 14-3-3sigma is a p53-regulated inhibitor of G2/M progression. Mol. Cell.

[57] Narita M., Nunez S., Heard E., Narita M., Lin A.W., Hearn S.A., Spector D.L., Hannon G.J., Lowe S.W. (2003). Rb-mediated heterochromatin formation and silencing of E2F target genes during cellular senescence. Cell.

[58] Xue W., Zender L., Miething C., Dickins R.A., Hernando E., Krizhanovsky V., Cordon-Cardo C., Lowe S.W. (2007). Senescence and tumour clearance is triggered by p53 restoration in murine liver carcinomas. Nature.

[59] Riley T., Sontag E., Chen P., Levine A. (2008). Transcriptional control of human p53-regulated genes. Nat. Rev. Mol. Cell Biol..

[60] Moll U.M., Marchenko N., Zhang X.K. (2006). p53 and Nur77/TR3—Transcription factors that directly target mitochondria for cell death induction. Oncogene.

[61] Green D.R., Kroemer G. (2009). Cytoplasmic functions of the tumour suppressor p53. Nature.

[62] Crighton D., Wilkinson S., O’Prey J., Syed N., Smith P., Harrison P.R., Gasco M., Garrone O., Crook T., Ryan K.M. (2006). DRAM, a p53-induced modulator of autophagy, is critical for apoptosis. Cell.

[63] Van Meir E.G., Polverini P.J., Chazin V.R., Su Huang H.J., de Tribolet N., Cavenee W.K. (1994). Release of an inhibitor of angiogenesis upon induction of wild type p53 expression in glioblastoma cells. Nat. Genet..

[64] Lin C., Liang Y., Zhu H., Zhang J., Zhong X. (2012). R280T mutation of p53 gene promotes proliferation of human glioma cells through GSK-3beta/PTEN pathway. Neurosci. Lett..

[65] Fontemaggi G., Dell’Orso S., Trisciuoglio D., Shay T., Melucci E., Fazi F., Terrenato I., Mottolese M., Muti P., Domany E. (2009). The execution of the transcriptional axis mutant p53, E2F1 and ID4 promotes tumor neo-angiogenesis. Nat. Struct. Mol. Biol..

[66] Ham S.W., Jeon H.Y., Jin X., Kim E.J., Kim J.K., Shin Y.J., Lee Y., Kim S.H., Lee S.Y., Seo S. (2019). TP53 gain-of-function mutation promotes inflammation in glioblastoma. Cell Death Differ..

[67] Qian X., Li X., Shi Z., Xia Y., Cai Q., Xu D., Tan L., Du L., Zheng Y., Zhao D. (2019). PTEN Suppresses Glycolysis by Dephosphorylating and Inhibiting Autophosphorylated PGK1. Mol. Cell.

[68] Lopez G., Noale M., Corti C., Gaudioso G., Sajjadi E., Venetis K., Gambini D., Runza L., Costanza J., Pesenti C. (2020). PTEN Expression as a Complementary Biomarker for Mismatch Repair Testing in Breast Cancer. Int. J. Mol. Sci..

[69] Mondin V.E., Ben El Kadhi K., Cauvin C., Jackson-Crawford A., Belanger E., Decelle B., Salomon R., Lowe M., Echard A., Carreno S. (2019). PTEN reduces endosomal PtdIns(4,5)P2 in a phosphatase-independent manner via a PLC pathway. J. Cell Biol..

[70] Shin Y.J., Sa J.K., Lee Y., Kim D., Chang N., Cho H.J., Son M., Oh M.Y.T., Shin K., Lee J.K. (2019). PIP4K2A as a negative regulator of PI3K in PTEN-deficient glioblastoma. J. Exp. Med..

[71] Benitez J.A., Ma J., D’Antonio M., Boyer A., Camargo M.F., Zanca C., Kelly S., Khodadadi-Jamayran A., Jameson N.M., Andersen M. (2017). PTEN regulates glioblastoma oncogenesis through chromatin-associated complexes of DAXX and histone H3.3. Nat. Commun..

[72] Yu Y., Xiong Y., Ladeiras D., Yang Z., Ming X.F. (2019). Myosin 1b Regulates Nuclear AKT Activation by Preventing Localization of PTEN in the Nucleus. iScience.

[73] Chung J.H., Ostrowski M.C., Romigh T., Minaguchi T., Waite K.A., Eng C. (2006). The ERK1/2 pathway modulates nuclear PTEN-mediated cell cycle arrest by cyclin D1 transcriptional regulation. Hum. Mol. Genet..

[74] Ge M.K., Zhang N., Xia L., Zhang C., Dong S.S., Li Z.M., Ji Y., Zheng M.H., Sun J., Chen G.Q. (2020). FBXO22 degrades nuclear PTEN to promote tumorigenesis. Nat. Commun..

[75] Han F., Hu R., Yang H., Liu J., Sui J., Xiang X., Wang F., Chu L., Song S. (2016). PTEN gene mutations correlate to poor prognosis in glioma patients: A meta-analysis. OncoTargets Ther..

[76] Chen Q., Weng H.Y., Tang X.P., Lin Y., Yuan Y., Li Q., Tang Z., Wu H.B., Yang S., Li Y. (2019). ARL4C stabilized by AKT/mTOR pathway promotes the invasion of PTEN-deficient primary human glioblastoma. J. Pathol..

[77] Chen P., Zhao D., Li J., Liang X., Li J., Chang A., Henry V.K., Lan Z., Spring D.J., Rao G. (2019). Symbiotic Macrophage-Glioma Cell Interactions Reveal Synthetic Lethality in PTEN-Null Glioma. Cancer Cell.

[78] Peng W., Chen J.Q., Liu C., Malu S., Creasy C., Tetzlaff M.T., Xu C., McKenzie J.A., Zhang C., Liang X. (2016). Loss of PTEN Promotes Resistance to T Cell-Mediated Immunotherapy. Cancer Discov..

[79] Kim H.S., Jang S.W., Lee W., Kim K., Sohn H., Hwang S.S., Lee G.R. (2017). PTEN drives Th17 cell differentiation by preventing IL-2 production. J. Exp. Med..

[80] Zhu Y., Guignard F., Zhao D., Liu L., Burns D.K., Mason R.P., Messing A., Parada L.F. (2005). Early inactivation of p53 tumor suppressor gene cooperating with NF1 loss induces malignant astrocytoma. Cancer Cell.

[81] Dischinger P.S., Tovar E.A., Essenburg C.J., Madaj Z.B., Gardner E.E., Callaghan M.E., Turner A.N., Challa A.K., Kempston T., Eagleson B. (2018). NF1 deficiency correlates with estrogen receptor signaling and diminished survival in breast cancer. NPJ Breast Cancer.

[82] Arima Y., Hayashi H., Kamata K., Goto T.M., Sasaki M., Kuramochi A., Saya H. (2010). Decreased expression of neurofibromin contributes to epithelial-mesenchymal transition in neurofibromatosis type 1. Exp. Dermatol..

[83] Cancer Genome Atlas Research N. (2008). Comprehensive genomic characterization defines human glioblastoma genes and core pathways. Nature.

[84] Herting C.J., Chen Z., Pitter K.L., Szulzewsky F., Kaffes I., Kaluzova M., Park J.C., Cimino P.J., Brennan C., Wang B. (2017). Genetic driver mutations define the expression signature and microenvironmental composition of high-grade gliomas. Glia.

[85] Alcantara Llaguno S.R., Wang Z., Sun D., Chen J., Xu J., Kim E., Hatanpaa K.J., Raisanen J.M., Burns D.K., Johnson J.E. (2015). Adult Lineage-Restricted CNS Progenitors Specify Distinct Glioblastoma Subtypes. Cancer Cell.

[86] Fadhlullah S.F.B., Halim N.B.A., Yeo J.Y.T., Ho R.L.Y., Um P., Ang B.T., Tang C., Ng W.H., Virshup D.M., Ho I.A.W. (2019). Pathogenic mutations in neurofibromin identifies a leucine-rich domain regulating glioma cell invasiveness. Oncogene.

[87] Wang Q., Hu B., Hu X., Kim H., Squatrito M., Scarpace L., deCarvalho A.C., Lyu S., Li P., Li Y. (2017). Tumor Evolution of Glioma-Intrinsic Gene Expression Subtypes Associates with Immunological Changes in the Microenvironment. Cancer Cell.

[88] Lu W., Zhang Y., Liu D., Songyang Z., Wan M. (2013). Telomeres-structure, function, and regulation. Exp. Cell Res..

[89] Jiang H., Ju Z., Rudolph K.L. (2007). Telomere shortening and ageing. Z. Gerontol. Geriatr..

[90] Leao R., Apolonio J.D., Lee D., Figueiredo A., Tabori U., Castelo-Branco P. (2018). Mechanisms of human telomerase reverse transcriptase (hTERT) regulation: Clinical impacts in cancer. J. Biomed. Sci..

[91] Meyerson M., Counter C.M., Eaton E.N., Ellisen L.W., Steiner P., Caddle S.D., Ziaugra L., Beijersbergen R.L., Davidoff M.J., Liu Q. (1997). hEST2, the putative human telomerase catalytic subunit gene, is up-regulated in tumor cells and during immortalization. Cell.

[92] Cong Y.S., Wen J., Bacchetti S. (1999). The human telomerase catalytic subunit hTERT: Organization of the gene and characterization of the promoter. Hum. Mol. Genet..

[93] Yuan X., Larsson C., Xu D. (2019). Mechanisms underlying the activation of TERT transcription and telomerase activity in human cancer: Old actors and new players. Oncogene.

[94] Castelo-Branco P., Choufani S., Mack S., Gallagher D., Zhang C., Lipman T., Zhukova N., Walker E.J., Martin D., Merino D. (2013). Methylation of the TERT promoter and risk stratification of childhood brain tumours: An integrative genomic and molecular study. Lancet Oncol..

[95] Barthel F.P., Wei W., Tang M., Martinez-Ledesma E., Hu X., Amin S.B., Akdemir K.C., Seth S., Song X., Wang Q. (2017). Systematic analysis of telomere length and somatic alterations in 31 cancer types. Nat. Genet..

[96] Killela P.J., Reitman Z.J., Jiao Y., Bettegowda C., Agrawal N., Diaz L.A., Friedman A.H., Friedman H., Gallia G.L., Giovanella B.C. (2013). TERT promoter mutations occur frequently in gliomas and a subset of tumors derived from cells with low rates of self-renewal. Proc. Natl. Acad. Sci. USA.

[97] Wang X., Li X., Xu F., Zhang Y., Liu H., Tao Y. (2016). Association of Telomerase Reverse Transcriptase Promoter Mutations with the Prognosis of Glioma Patients: A Meta-Analysis. Mol. Neurobiol..

[98] Huang F.W., Hodis E., Xu M.J., Kryukov G.V., Chin L., Garraway L.A. (2013). Highly recurrent TERT promoter mutations in human melanoma. Science.

[99] Watts C., Price S.J., Santarius T. (2014). Current concepts in the surgical management of glioma patients. Clin. Oncol..

[100] Fernandes C., Costa A., Osorio L., Lago R.C., Linhares P., Carvalho B., Caeiro C., De Vleeschouwer S. (2017). Current standards of care in glioblastoma therapy. Glioblastoma.

[101] Stummer W., Nestler U., Stockhammer F., Krex D., Kern B.C., Mehdorn H.M., Vince G.H., Pichlmeier U. (2011). Favorable outcome in the elderly cohort treated by concomitant temozolomide radiochemotherapy in a multicentric phase II safety study of 5-ALA. J. Neurooncol..

[102] De Witt Hamer P.C., Robles S.G., Zwinderman A.H., Duffau H., Berger M.S. (2012). Impact of intraoperative stimulation brain mapping on glioma surgery outcome: A meta-analysis. J. Clin. Oncol..

[103] Pallud J., Rigaux-Viode O., Corns R., Muto J., Lopez Lopez C., Mellerio C., Sauvageon X., Dezamis E. (2017). Direct electrical bipolar electrostimulation for functional cortical and subcortical cerebral mapping in awake craniotomy. Practical considerations. Neurochirurgie.

[104] Mueller W.M., Yetkin F.Z., Hammeke T.A., Morris G.L., Swanson S.J., Reichert K., Cox R., Haughton V.M. (1996). Functional magnetic resonance imaging mapping of the motor cortex in patients with cerebral tumors. Neurosurgery.

[105] Lacroix M., Abi-Said D., Fourney D.R., Gokaslan Z.L., Shi W., DeMonte F., Lang F.F., McCutcheon I.E., Hassenbusch S.J., Holland E. (2001). A multivariate analysis of 416 patients with glioblastoma multiforme: Prognosis, extent of resection, and survival. J. Neurosurg..

[106] Sanai N., Polley M.Y., McDermott M.W., Parsa A.T., Berger M.S. (2011). An extent of resection threshold for newly diagnosed glioblastomas. J. Neurosurg..

[107] Keime-Guibert F., Chinot O., Taillandier L., Cartalat-Carel S., Frenay M., Kantor G., Guillamo J.S., Jadaud E., Colin P., Bondiau P.Y. (2007). Radiotherapy for glioblastoma in the elderly. N. Engl. J. Med..

[108] Roa W., Brasher P.M., Bauman G., Anthes M., Bruera E., Chan A., Fisher B., Fulton D., Gulavita S., Hao C. (2004). Abbreviated course of radiation therapy in older patients with glioblastoma multiforme: A prospective randomized clinical trial. J. Clin. Oncol..

[109] Stupp R., Mason W.P., van den Bent M.J., Weller M., Fisher B., Taphoorn M.J., Belanger K., Brandes A.A., Marosi C., Bogdahn U. (2005). Radiotherapy plus concomitant and adjuvant temozolomide for glioblastoma. N. Engl. J. Med..

[110] Strobel H., Baisch T., Fitzel R., Schilberg K., Siegelin M.D., Karpel-Massler G., Debatin K.M., Westhoff M.A. (2019). Temozolomide and Other Alkylating Agents in Glioblastoma Therapy. Biomedicines.

[111] Aasland D., Gotzinger L., Hauck L., Berte N., Meyer J., Effenberger M., Schneider S., Reuber E.E., Roos W.P., Tomicic M.T. (2019). Temozolomide Induces Senescence and Repression of DNA Repair Pathways in Glioblastoma Cells via Activation of ATR-CHK1, p21, and NF-kappaB. Cancer Res..

[112] Portnow J., Badie B., Chen M., Liu A., Blanchard S., Synold T.W. (2009). The neuropharmacokinetics of temozolomide in patients with resectable brain tumors: Potential implications for the current approach to chemoradiation. Clin. Cancer Res..

[113] Cohen M.H., Johnson J.R., Pazdur R. (2005). Food and Drug Administration Drug approval summary: Temozolomide plus radiation therapy for the treatment of newly diagnosed glioblastoma multiforme. Clin. Cancer Res..

[114] Baker S.D., Wirth M., Statkevich P., Reidenberg P., Alton K., Sartorius S.E., Dugan M., Cutler D., Batra V., Grochow L.B. (1999). Absorption, metabolism, and excretion of 14C-temozolomide following oral administration to patients with advanced cancer. Clin. Cancer Res..

[115] Quinn J.A., Jiang S.X., Reardon D.A., Desjardins A., Vredenburgh J.J., Rich J.N., Gururangan S., Friedman A.H., Bigner D.D., Sampson J.H. (2009). Phase II trial of temozolomide plus o6-benzylguanine in adults with recurrent, temozolomide-resistant malignant glioma. J. Clin. Oncol..

[116] Lee C.Y. (2017). Strategies of temozolomide in future glioblastoma treatment. OncoTargets Ther..

[117] Fang C., Wang K., Stephen Z.R., Mu Q., Kievit F.M., Chiu D.T., Press O.W., Zhang M. (2015). Temozolomide nanoparticles for targeted glioblastoma therapy. ACS Appl. Mater. Interfaces.

[118] Esteller M., Garcia-Foncillas J., Andion E., Goodman S.N., Hidalgo O.F., Vanaclocha V., Baylin S.B., Herman J.G. (2000). Inactivation of the DNA-repair gene MGMT and the clinical response of gliomas to alkylating agents. N. Engl. J. Med..

[119] Hermisson M., Klumpp A., Wick W., Wischhusen J., Nagel G., Roos W., Kaina B., Weller M. (2006). O6-methylguanine DNA methyltransferase and p53 status predict temozolomide sensitivity in human malignant glioma cells. J. Neurochem..

[120] Friedman H.S., McLendon R.E., Kerby T., Dugan M., Bigner S.H., Henry A.J., Ashley D.M., Krischer J., Lovell S., Rasheed K. (1998). DNA mismatch repair and O6-alkylguanine-DNA alkyltransferase analysis and response to Temodal in newly diagnosed malignant glioma. J. Clin. Oncol..

[121] Warde-Farley D., Donaldson S.L., Comes O., Zuberi K., Badrawi R., Chao P., Franz M., Grouios C., Kazi F., Lopes C.T. (2010). The GeneMANIA prediction server: Biological network integration for gene prioritization and predicting gene function. Nucleic Acids Res..

[122] Hegi M.E., Diserens A.C., Gorlia T., Hamou M.F., de Tribolet N., Weller M., Kros J.M., Hainfellner J.A., Mason W., Mariani L. (2005). MGMT gene silencing and benefit from temozolomide in glioblastoma. N. Engl. J. Med..

[123] Chen X., Zhang M., Gan H., Wang H., Lee J.H., Fang D., Kitange G.J., He L., Hu Z., Parney I.F. (2018). A novel enhancer regulates MGMT expression and promotes temozolomide resistance in glioblastoma. Nat. Commun..

[124] Broniscer A., Chintagumpala M., Fouladi M., Krasin M.J., Kocak M., Bowers D.C., Iacono L.C., Merchant T.E., Stewart C.F., Houghton P.J. (2006). Temozolomide after radiotherapy for newly diagnosed high-grade glioma and unfavorable low-grade glioma in children. J. Neurooncol..

[125] Agnihotri S., Burrell K., Buczkowicz P., Remke M., Golbourn B., Chornenkyy Y., Gajadhar A., Fernandez N.A., Clarke I.D., Barszczyk M.S. (2014). ATM regulates 3-methylpurine-DNA glycosylase and promotes therapeutic resistance to alkylating agents. Cancer Discov..

[126] Gaspar N., Marshall L., Perryman L., Bax D.A., Little S.E., Viana-Pereira M., Sharp S.Y., Vassal G., Pearson A.D., Reis R.M. (2010). MGMT-independent temozolomide resistance in pediatric glioblastoma cells associated with a PI3-kinase-mediated HOX/stem cell gene signature. Cancer Res..

[127] Maiuri M.C., Kroemer G. (2015). Autophagy in stress and disease. Cell Death Differ..

[128] Eskelinen E.L. (2019). Autophagy: Supporting cellular and organismal homeostasis by self-eating. Int. J. Biochem. Cell Biol..

[129] Kanzawa T., Germano I.M., Komata T., Ito H., Kondo Y., Kondo S. (2004). Role of autophagy in temozolomide-induced cytotoxicity for malignant glioma cells. Cell Death Differ..

[130] Shen X., Kan S., Hu J., Li M., Lu G., Zhang M., Zhang S., Hou Y., Chen Y., Bai Y. (2016). EMC6/TMEM93 suppresses glioblastoma proliferation by modulating autophagy. Cell Death Dis..

[131] Das S., Marsden P.A. (2013). Angiogenesis in glioblastoma. N. Engl. J. Med..

[132] Folkman J. (1971). Tumor angiogenesis: Therapeutic implications. N. Engl. J. Med..

[133] Wang R., Chadalavada K., Wilshire J., Kowalik U., Hovinga K.E., Geber A., Fligelman B., Leversha M., Brennan C., Tabar V. (2010). Glioblastoma stem-like cells give rise to tumour endothelium. Nature.

[134] Soda Y., Marumoto T., Friedmann-Morvinski D., Soda M., Liu F., Michiue H., Pastorino S., Yang M., Hoffman R.M., Kesari S. (2011). Transdifferentiation of glioblastoma cells into vascular endothelial cells. Proc. Natl. Acad. Sci. USA.

[135] Greenblatt M., Shubi P. (1968). Tumor angiogenesis: Transfilter diffusion studies in the hamster by the transparent chamber technique. J. Natl. Cancer Inst..

[136] Reardon D.A., Wen P.Y., Desjardins A., Batchelor T.T., Vredenburgh J.J. (2008). Glioblastoma multiforme: An emerging paradigm of anti-VEGF therapy. Expert Opin. Biol. Ther..

[137] Hurwitz H., Fehrenbacher L., Novotny W., Cartwright T., Hainsworth J., Heim W., Berlin J., Baron A., Griffing S., Holmgren E. (2004). Bevacizumab plus irinotecan, fluorouracil, and leucovorin for metastatic colorectal cancer. N. Engl. J. Med..

[138] Al-Husein B., Abdalla M., Trepte M., Deremer D.L., Somanath P.R. (2012). Antiangiogenic therapy for cancer: An update. Pharmacotherapy.

[139] Chinot O.L., Wick W., Mason W., Henriksson R., Saran F., Nishikawa R., Carpentier A.F., Hoang-Xuan K., Kavan P., Cernea D. (2014). Bevacizumab plus radiotherapy-temozolomide for newly diagnosed glioblastoma. N. Engl. J. Med..

[140] Gilbert M.R., Dignam J.J., Armstrong T.S., Wefel J.S., Blumenthal D.T., Vogelbaum M.A., Colman H., Chakravarti A., Pugh S., Won M. (2014). A randomized trial of bevacizumab for newly diagnosed glioblastoma. N. Engl. J. Med..

[141] Hainsworth J.D., Becker K.P., Mekhail T., Chowdhary S.A., Eakle J.F., Wright D., Langdon R.M., Yost K.J., Padula G.D.A., West-Osterfield K. (2019). Phase I/II study of bevacizumab with BKM120, an oral PI3K inhibitor, in patients with refractory solid tumors (phase I) and relapsed/refractory glioblastoma (phase II). J. Neurooncol..

[142] Galanis E., Anderson S.K., Twohy E.L., Carrero X.W., Dixon J.G., Tran D.D., Jeyapalan S.A., Anderson D.M., Kaufmann T.J., Feathers R.W. (2019). A phase 1 and randomized, placebo-controlled phase 2 trial of bevacizumab plus dasatinib in patients with recurrent glioblastoma: Alliance/North Central Cancer Treatment Group N0872. Cancer.

[143] Hata N., Mizoguchi M., Kuga D., Hatae R., Akagi Y., Sangatsuda Y., Amemiya T., Michiwaki Y., Fujioka Y., Takigawa K. (2020). First-line bevacizumab contributes to survival improvement in glioblastoma patients complementary to temozolomide. J. Neurooncol..

[144] Rotte A. (2019). Combination of CTLA-4 and PD-1 blockers for treatment of cancer. J. Exp. Clin. Cancer Res..

[145] Sharma P., Allison J.P. (2015). Immune checkpoint targeting in cancer therapy: Toward combination strategies with curative potential. Cell.

[146] Parsa A.T., Waldron J.S., Panner A., Crane C.A., Parney I.F., Barry J.J., Cachola K.E., Murray J.C., Tihan T., Jensen M.C. (2007). Loss of tumor suppressor PTEN function increases B7-H1 expression and immunoresistance in glioma. Nat. Med..

[147] Wang X., Guo G., Guan H., Yu Y., Lu J., Yu J. (2019). Challenges and potential of PD-1/PD-L1 checkpoint blockade immunotherapy for glioblastoma. J. Exp. Clin. Cancer Res..

[148] Reardon D.A., Brandes A.A., Omuro A., Mulholland P., Lim M., Wick A., Baehring J., Ahluwalia M.S., Roth P., Bahr O. (2020). Effect of Nivolumab vs. Bevacizumab in Patients with Recurrent Glioblastoma: The CheckMate 143 Phase 3 Randomized Clinical Trial. JAMA Oncol..

[149] Omuro A., Vlahovic G., Lim M., Sahebjam S., Baehring J., Cloughesy T., Voloschin A., Ramkissoon S.H., Ligon K.L., Latek R. (2018). Nivolumab with or without ipilimumab in patients with recurrent glioblastoma: Results from exploratory phase I cohorts of CheckMate 143. Neuro-Oncology.

[150] Cobbs C.S., Harkins L., Samanta M., Gillespie G.Y., Bharara S., King P.H., Nabors L.B., Cobbs C.G., Britt W.J. (2002). Human cytomegalovirus infection and expression in human malignant glioma. Cancer Res..

[151] Prins R.M., Cloughesy T.F., Liau L.M. (2008). Cytomegalovirus immunity after vaccination with autologous glioblastoma lysate. N. Engl. J. Med..

[152] Mitchell D.A., Batich K.A., Gunn M.D., Huang M.N., Sanchez-Perez L., Nair S.K., Congdon K.L., Reap E.A., Archer G.E., Desjardins A. (2015). Tetanus toxoid and CCL3 improve dendritic cell vaccines in mice and glioblastoma patients. Nature.

[153] Batich K.A., Reap E.A., Archer G.E., Sanchez-Perez L., Nair S.K., Schmittling R.J., Norberg P., Xie W., Herndon J.E., Healy P. (2017). Long-term Survival in Glioblastoma with Cytomegalovirus pp65-Targeted Vaccination. Clin. Cancer Res..

[154] Batich K.A., Mitchell D.A., Healy P., Herndon J.E., Sampson J.H. (2020). Once, Twice, Three Times a Finding: Reproducibility of Dendritic Cell Vaccine Trials Targeting Cytomegalovirus in Glioblastoma. Clin. Cancer Res..

[155] Lun X., Wells J.C., Grinshtein N., King J.C., Hao X., Dang N.H., Wang X., Aman A., Uehling D., Datti A. (2016). Disulfiram when Combined with Copper Enhances the Therapeutic Effects of Temozolomide for the Treatment of Glioblastoma. Clin. Cancer Res..

[156] Teng J., Hejazi S., Hiddingh L., Carvalho L., de Gooijer M.C., Wakimoto H., Barazas M., Tannous M., Chi A.S., Noske D.P. (2018). Recycling drug screen repurposes hydroxyurea as a sensitizer of glioblastomas to temozolomide targeting de novo DNA synthesis, irrespective of molecular subtype. Neuro-Oncology.

[157] Murphy S.F., Varghese R.T., Lamouille S., Guo S., Pridham K.J., Kanabur P., Osimani A.M., Sharma S., Jourdan J., Rodgers C.M. (2016). Connexin 43 Inhibition Sensitizes Chemoresistant Glioblastoma Cells to Temozolomide. Cancer Res..

[158] Goellner E.M., Grimme B., Brown A.R., Lin Y.C., Wang X.H., Sugrue K.F., Mitchell L., Trivedi R.N., Tang J.B., Sobol R.W. (2011). Overcoming temozolomide resistance in glioblastoma via dual inhibition of NAD+ biosynthesis and base excision repair. Cancer Res..

[159] Aldous A.R., Dong J.Z. (2018). Personalized neoantigen vaccines: A new approach to cancer immunotherapy. Bioorg. Med. Chem..

[160] Ott P.A., Hu Z., Keskin D.B., Shukla S.A., Sun J., Bozym D.J., Zhang W., Luoma A., Giobbie-Hurder A., Peter L. (2017). An immunogenic personal neoantigen vaccine for patients with melanoma. Nature.

[161] Hacohen N., Fritsch E.F., Carter T.A., Lander E.S., Wu C.J. (2013). Getting personal with neoantigen-based therapeutic cancer vaccines. Cancer Immunol. Res..

[162] Hilf N., Kuttruff-Coqui S., Frenzel K., Bukur V., Stevanovic S., Gouttefangeas C., Platten M., Tabatabai G., Dutoit V., van der Burg S.H. (2019). Actively personalized vaccination trial for newly diagnosed glioblastoma. Nature.

[163] Keskin D.B., Anandappa A.J., Sun J., Tirosh I., Mathewson N.D., Li S., Oliveira G., Giobbie-Hurder A., Felt K., Gjini E. (2019). Neoantigen vaccine generates intratumoral T cell responses in phase Ib glioblastoma trial. Nature.

[164] Aurisicchio L., Pallocca M., Ciliberto G., Palombo F. (2018). The perfect personalized cancer therapy: Cancer vaccines against neoantigens. J. Exp. Clin. Cancer Res..

